# Antioxidants in Plants: A Valorization Potential Emphasizing the Need for the Conservation of Plant Biodiversity in Cuba

**DOI:** 10.3390/antiox9111048

**Published:** 2020-10-27

**Authors:** Gabriel Llauradó Maury, Daniel Méndez Rodríguez, Sophie Hendrix, Julio César Escalona Arranz, Yilan Fung Boix, Ania Ochoa Pacheco, Jesús García Díaz, Humberto J. Morris-Quevedo, Albys Ferrer Dubois, Elizabeth Isaac Aleman, Natalie Beenaerts, Isidro E. Méndez-Santos, Teresa Orberá Ratón, Paul Cos, Ann Cuypers

**Affiliations:** 1 Centre of Studies for Industrial Biotechnology (CEBI), University of Oriente, Avenida Patricio Lumumba s/n, Reparto Jiménez, Santiago de Cuba CP 90500, Cuba; gabriel@uo.edu.cu (G.L.M.); jquevedo@uo.edu.cu (H.J.M.-Q.); torbera@uo.edu.cu (T.O.R.); 2 Faculty of Applied Sciences, University of Camagüey, Carretera Circunvalación Norte, km 5 ½, Camagüey CP 70100, Cuba; daniel.mendez@reduc.edu.cu (D.M.R.); isidro.mendez@reduc.edu.cu (I.E.M.-S.); 3 Centre for Environmental Sciences, Campus Diepenbeek, Hasselt University, Agoralaan Building D, BE-3590 Diepenbeek, Belgium; sophie.hendrix@uhasselt.be (S.H.); natalie.beenaerts@uhasselt.be (N.B.); 4 Laboratory for Microbiology, Parasitology and Hygiene (LMPH), University of Antwerp, Universiteitsplein 1, BE-2610 Antwerp, Belgium; 5 Pharmacy Department, University of Oriente, Avenida Patricio Lumumba s/n, Reparto Jiménez, Santiago de Cuba CP 90500, Cuba; jcea@uo.edu.cu (J.C.E.A.); aochoap@uo.edu.cu (A.O.P.); jgadi@uo.edu.cu (J.G.D.); 6 National Center of Applied Electromagnetism, University of Oriente, Avenida Las Américas s/n, P.O. Box 4078, Santiago de Cuba CP 90400, Cuba; yilan@uo.edu.cu (Y.F.B.); albys@uo.edu.cu (A.F.D.); elizabetha@uo.edu.cu (E.I.A.)

**Keywords:** antioxidants, secondary metabolites, Cuba, plant biodiversity, plant protection, crop production, human health, nutraceutical, pharmaceutical

## Abstract

Plants are phytochemical hubs containing antioxidants, essential for normal plant functioning and adaptation to environmental cues and delivering beneficial properties for human health. Therefore, knowledge on the antioxidant potential of different plant species and their nutraceutical and pharmaceutical properties is of utmost importance. Exploring this scientific research field provides fundamental clues on (1) plant stress responses and their adaptive evolution to harsh environmental conditions and (2) (new) natural antioxidants with a functional versatility to prevent and treat human pathologies. These natural antioxidants can be valorized via plant-derived foods and products. Cuba contains an enormously rich plant biodiversity harboring a great antioxidant potential. Besides opening new avenues for the implementation of sustainable agroecological practices in crop production, it will also contribute to new strategies to preserve plant biodiversity and simultaneously improve nature management policies in Cuba. This review provides an overview on the beneficial properties of antioxidants for plant protection and human health and is directed to the valorization of these plant antioxidants, emphasizing the need for biodiversity conservation.

## 1. Introduction

Plant antioxidants are a natural reservoir of bioactive compounds. They play important roles in plant acclimation and adaptation to environmental challenges, but are also beneficial for human health. As sedentary organisms, plants cannot escape from environmental challenges, originating from natural origin (e.g., temperature, water availability, soil composition, pests…) or from anthropogenic practices (e.g., destruction of habitats, pollution…). Diverse abiotic factors, like pollution as well as nutrient deficiency, temperature regimes (heat/cold), water supply (drought/flooding), light intensity, day/night rhythms, and radiation, modify the balance between production and scavenging of reactive oxygen species (ROS) and induce a phenomenon well known as oxidative stress [1,2,3,4]. Although ROS are crucial for normal plant growth and development, and play important roles in signal transduction, they are also able to induce cellular damage [5,6,7]. Therefore, maintaining the oxidative balance is crucial for plant stress adaptation.

To prevent oxidative damage, plants possess an extensive antioxidant defense system consisting of enzymes and metabolites. Ascorbate (AsA, vitamin C) and glutathione (GSH) are the major water-soluble antioxidant metabolites, but also secondary metabolites, such as polyphenols, flavonoids and terpenoids, participate in the detoxification of ROS under different environmental stresses [4,8,9,10,11]. Many of these plant secondary metabolites also display biological activity against insects, fungi and other microorganisms [12,13], forming the basis for their medicinal use. Both, the quantity and quality of secondary metabolites are determined by intrinsic plant characteristics (e.g., developmental stage) on one hand and the environment on the other hand [14,15]. As environmental factors can affect the production of antioxidants and secondary metabolites, this in turn affects the plant’s nutritional and medicinal value for human health [16,17].

A plethora of evidence validates that antioxidants present in plant-derived products and foods encompass many biomedical applications. These antioxidants play a role in the prevention as well as in the complementary treatment of non-infectious chronic diseases, such as cardiovascular, inflammatory and neurodegenerative disorders, metabolic syndrome and cancer [18,19,20,21,22]. Antioxidants are classified in different categories, but low-molecular weight compounds like terpenoids, alkaloids and mainly polyphenols gained a lot of interest. Polyphenols represent a variety of pharmacologically active phytochemicals that are investigated mainly because of their ability to delay or inhibit oxidation processes as a consequence of some cellular pathological conditions [23,24].

As phytochemical hubs, plants also contain a significant number of nutrients important for health promotion. The use of the undeniable nutritional value of several plants rich in nutrients, vitamins, minerals, and also phenolic compounds in the design of new formulations and promising industrial products is an attractive approach. These bioproducts have been termed as functional foods, nutraceutical, cosmeceutical and dietary supplements [25,26,27], known to be at the interface between nutrition and medicine [28,29].

Over the last decade, there has been a growing research interest in these natural antioxidants (e.g., phenolic antioxidants, vitamins…) because of their overall safety and beneficial properties [26,27,30,31]. Latin America and the Caribbean regions harbor a large number of plants containing antioxidant potential [32,33,34,35,36]. However, the biodiversity of these plants is largely unknown and therefore underexploited. Furthermore, this region is highly affected by global change and habitat loss. Therefore, these (endemic) plants might face extinction without being investigated for their antioxidant value.

Cuba is a unique case as it faces specific environmental challenges, such as extreme temperatures, drought stress, soil conditions…, but it also contains an enormously rich (endemic) plant biodiversity [37,38,39]. Understanding the correlation between antioxidant compounds in non-model plants and their evolutionary advantage to withstand specific stress conditions should be included in strategies to protect plant biodiversity. Moreover, the antioxidant spectrum present in Cuban plant species should be maintained via sustainable agroecological practices.

This review provides an overview on the antioxidant potential in Cuban plant biodiversity in relation to human health and plant stress adaptation. Expanding our current knowledge on plant biodiversity and its antioxidant potential might support the conservation of plant biodiversity and hence preservation of endangered species as well as open new avenues for plant/crop production for agronomical and industrial applications.

## 2. Cuba and Its Endemic Potentials

The Republic of Cuba is an archipelago with more than 1600 islands, islets and cays. Its territorial extension amounts to 109,880 km^2^ (FAO, 2015). Around 20,800 terrestrial species have been identified, of which 8948 (43%) are endemics [38]. Cuba is recognized as the island with the highest degree of endemism in the West-Indies, including more than half of its plant species [40]. Islands in general contain a higher degree of endemism compared to the mainland, but threats such as land cover loss, human activities and insufficient protection measures should be considered in time [41]. In a recent study, about 67% of native taxa were evaluated and about half of them are now categorized as threatened. Alarmingly, about 65% of these threatened species are considered endemic [42]. The variety of different habitats and especially soils in Cuba might have enhanced speciation processes that occurred in relatively extreme conditions [43,44]. For example, speciation on (1) soils derived from serpentinized ultramafic rocks that are rich in magnesium and heavy metals, but poor in calcium and with high pH values [45]; (2) soils originating from rocks rich in quartz (quartz-alitic) and sand, which are poor in nutrients (in the case of the so-called white sands, markedly oligotrophic), have low water retention capacity and a rather acidic pH [46]; and (3) soils where bare karst more or less predominates, which is the case for the more recently emerged arid coasts and elevations with advanced carsification processes, especially mountains with a mogotiform appearance [47].

Due to its geographical location, Cuba is subject to a humid tropical climate, which might alter into a dry tropical climate due to climate change [48]. The dynamics of ecosystems can worsen this aridity due to the combined effect of increased drought, temperature and evaporation. The rise in mean sea level and other changes associated with the water regime, will not only cause the movement, fragmentation, alteration and destruction of certain habitats or even the disappearance of entire ecosystems, especially wetlands, but also favor salinity in aquifers and greater transfer of it to the soil [38]. In this context, physiological and biochemical modifications are expected in the flora due to stress processes, changes in phenology, phase shift between the biological cycles of plants in relation to their pollinators and biological controllers possibly leading to the extinction of several species.

Many Cuban species, endemic or not, have evolutionary adaptations because they had to tackle different harsh environmental conditions for a long time and therefore harbor unique genetic characteristics/features [30]. As such, they are more resilient to abiotic stresses as compared to their mainland sister species. It is known that the increase in various environmental stresses unbalances the production of ROS and limits the activity of antioxidants, causing oxidative damage. Therefore, a greater production of antioxidants helps the plant to resist environmental stress [49]. In addition, it has been shown that many of these species have a high phytochemical content considered beneficial to human health [30]. In Table 1, an overview is given of plants with antioxidant potential or plant-derived products that are frequently used by the Cuban population according to the “Basic List of Medications and Natural Products in Cuba” (Dirección de medicamentos y tecnologías médicas, 2018).

To document the overall impact of the antioxidant research in Cuba, specifically in plants and related fields, a bibliometric analysis was performed in July 2020. A brief literature search was performed using the Scopus online database (https://www.scopus.com/) to identify Cuban papers indexed in Scopus with the following keywords: ‘antioxidant’ (=antioxida∗; anti-oxida∗) and ‘plant’. This strategy searched for papers containing the aforementioned words and its derivatives in their title, abstract, or keywords. Thereafter, these manuscripts were imported into VOSviewer for a bibliometric analysis based on keywords [50]. This bibliometric analysis focused on 2 groups of topic-related manuscripts, i.e., (1) plant-related manuscripts and (2) antioxidant-related manuscripts (Appendix A). These analyses demonstrate that the majority of scientific work on Cuban plants (group 1) is related to medicinal plants, the chemical composition of plant extracts and that antioxidant properties are the main analyzed biological activity. Of the total number of publications (3056), 94 were specific to endemic species, of which *Phyllantus* and *Euphorbia* are the two most studied genera. Concerning the antioxidant-related investigations in Cuba (group 2), the topics are centered around oxidative stress, and the main investigated compounds are flavonoids, highlighting the presence of the phenolic compound mangiferin (mainly present in plants of the *Mangifera* genus). Of the total number of publications (699) in this second group, nine were specific to endemic species, showing that this type of plant is not well studied. Cuban flora is pharmacologically and chemically under-investigated, most probably because there are insufficient technological and human resources to isolate and evaluate metabolites of pharmacological and nutraceutical interest. Another problem is the collection of endemic plants from the wild, especially endangered species. Here, plant biotechnological techniques could offer a viable alternative.

## 3. Antioxidants in Plant Protection

**Figure 1:**
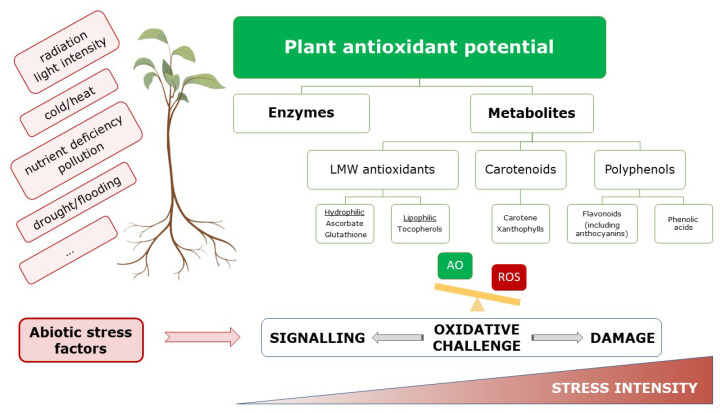
Plant antioxidant potential to maintain the cellular redox balance in order to cope with abiotic stress factors. AO = Antioxidants; ROS = Reactive Oxygen Species.

The environment of plants is ever-changing and involves variation of natural origin (e.g., circadian rhythm, seasons, temperature, physicochemical soil characteristics, radiation) as well as alterations resulting from anthropogenic activities (e.g., pollution and climate change). Due to their sessile nature, plants are more prone to these environmental challenges and are therefore often subject to stress. This translates into an oxidative challenge characterized by increased ROS production [5]. These ROS play crucial roles in oxidative signaling and, together with other components like plant hormones, initiate an immediate and effective response to ensure plant acclimation and eventually adaptation to a stressful environment [1]. Nevertheless, increased ROS concentrations can also result in damage to cellular macromolecules such as DNA, proteins and membrane lipids. In order to finetune ROS levels to prevent oxidative damage, but still allowing oxidative signaling, plants possess an extensive antioxidant defense system consisting of enzymatic and non-enzymatic components (Figure 1). Major enzymatic antioxidants are superoxide dismutase (SOD), catalase (CAT), peroxidases (POD) and members of the redoxin family [5]. Whereas these are directly involved in ROS detoxification, enzymes of the AsA-GSH cycle [monodehydroascorbate reductase (MDHAR); dehydroascorbate reductase (DHAR); glutathione reductase (GR)] and enzymes providing reducing power are important in recycling non-enzymatic antioxidants such as AsA and GSH. Both are important low molecular weight (LMW), water-soluble molecules [51].

### 3.1. Non-Enzymatic Antioxidants in Plants

Ascorbic acid is the most important substrate for H_2_O_2_ detoxification in plant cells. It functions as the electron donor for ascorbate peroxidase (APX), which reduces H_2_O_2_ to water. As a consequence, AsA is oxidized to monodehydroascorbic acid (MDHA), which can disproportionate into AsA and dehydroascorbic acid (DHA). Whereas MDHA is again reduced to AsA via MDHAR using electrons derived from NAPDH, DHA is converted to AsA via the action of DHAR. This enzyme uses GSH as an electron donor, causing its oxidation to glutathione disulfide (GSSG). This is reduced back to GSH by GR, using reducing equivalents derived from NAPDH. This metabolic pathway for the detoxification of H_2_O_2_ is referred to as the AsA-GSH cycle or Foyer-Halliwell-Asada pathway and functions in all subcellular compartments except vacuoles [52]. In addition to its role in this antioxidant pathway, AsA plays important roles in processes involved in normal plant development, such as cell division, cell wall expansion and cell elongation [53,54]. Furthermore, it is necessary for the biosynthesis of anthocyanins, flavonoids, glucosinolates and several phytohormones [55]. As such, AsA is a key player in plant protection against a wide range of abiotic stresses such as ozone, high light, drought and metals [52]. Glutathione—a tripeptide consisting of γ-glutamate, cysteine and glycine—constitutes the second major non-enzymatic antioxidant involved in ROS detoxification via the AsA-GSH cycle. In addition, GSH can directly scavenge hydroxyl radicals and singlet oxygen [56]. Besides its role in antioxidant defense, GSH plays additional roles in plant protection (1) by forming conjugates with xenobiotics via glutathione-S-transferases and (2) as a precursor for the synthesis of phytochelatins, which are involved in the chelation and sequestration of metals such as cadmium (Cd) and metalloids like arsenic (As) [57]. Besides these hydrophilic low molecular weight reductants, plants also possess lipophilic antioxidants. Among these, tocopherols (a type of vitamin E) play a crucial role in protecting cells from lipid peroxidation, which occurs when hydroxyl radicals attack polyunsaturated fatty acids in membranes. The antioxidant potential of tocopherols relies on their ability to scavenge the resulting lipid peroxyl radicals as well as singlet oxygen. Whereas four different forms of tocopherol exist (α, β, γ and δ), α-tocopherol harbors the highest biological activity and accounts for over 90% of the foliar vitamin E content [58].

As photosynthesis lies at the heart of plant functioning, protection of this process from the negative consequences of stress-induced ROS production is of utmost importance. Key players in safeguarding photosynthesis are carotenoids. The photoprotective role of these metabolites derives from their ability to quench excited chlorophyll states, scavenge ROS and dissipate excess energy as heat. As such, they are crucial for plant protection from high light stress [59,60]. Carotenoids are tetraterpenoid pigments characterized by yellow, orange, red and purple colors. In general, their structure consists of a polyene chain with nine conjugated double bonds and an end group at both ends. Carotenoids can be subdivided into two main groups: carotenes and xantophylls. Examples of carotenes are α-carotene, β-carotene, γ-carotene and lycopene. The group of xantophylls consists of more than 800 molecules, including zeaxanthin, lutein and peridinin [61]. Carotenoids are biosynthesized and stored in plastids. Besides their role in photosynthesis and photoprotection, carotenoids also fulfill other functions in plants. For example, they provide precursors for the biosynthesis of certain phytohormones and play a role as signaling molecules mediating plant development and responses to environmental cues [59,60].

Another important class of plant antioxidants are polyphenols, which are composed of aromatic rings with one or more hydroxyl groups. This class of secondary metabolites consists of more than 8000 compounds, which all derive from either phenylalanine or its precursor shikimic acid through the shikimate and phenylpropanoid pathways. Polyphenols are subdivided into several groups based on their number of phenol groups or the structural elements connecting the phenol groups. The most important polyphenol subclasses are phenolic acids, flavonoids, stilbenes and lignans. Phenolic acids are derivatives of either benzoic acid or cinnamic acid. The flavonoids class of polyphenols consists of over 4000 compounds and is subdivided into six groups: flavonols, flavones, flavanones, flavanols, isoflavones and anthocyanins [61]. Flavonoids are mainly found in the leaf epidermis and fruit skin [12]. Stilbenes generally act as antifungal compounds and are only synthesized in response to wounding or infection [62]. In general, polyphenols play an important role in plant growth and development, through their involvement in seed germination, cell division, signal transduction, hormonal regulation, regulation of photosynthetic activity and reproduction [10]. Furthermore, many polyphenols harbor antioxidant potential as a consequence of their ability to scavenge free radicals, donate hydrogen atoms or electrons and chelate metal cations [63]. In response to abiotic stresses, biosynthesis of polyphenols is stimulated in plants and enhances their resistance. Flavonoids, for example, contribute to plant protection from UV light, whereas anthocyanins are involved in resistance to visible light [10].

### 3.2. Antioxidants in Plants under Abiotic Stress Conditions

To prevent oxidative damage as a consequence of increased ROS production, the activity and biosynthesis of plant antioxidants are generally increased in response to stress conditions. Interestingly, the ability to combat oxidative stress by enhancing antioxidant enzyme activities and biosynthesis of antioxidant metabolites plays a key role in defining plant stress tolerance and differs between sensitive and tolerant cultivars of the same species. The following paragraphs describe the impact of abiotic stress factors on several crops frequently cultivated in Cuba.

Dharshini et al., (2020) demonstrated that low-temperature stress increases the activity of SOD, CAT and POD in roots of sugarcane (*Saccharum spontaneum*). Furthermore, transcriptional profiling revealed that cold stress enhanced the expression levels of several genes involved in carotenoid biosynthesis and the phenylpropanoid pathway, suggesting an increased production of carotenoids and phenolic compounds such as flavonoids, p-hydroxyphenyl lignin and coniferin in sugarcane roots [64]. Whereas chilling stress did not affect AsA and GSH concentrations, it significantly enhanced AsA/DHA and GSH/GSSG ratios in leaves of *Saccharum officinarum*, another sugarcane species. This response coincided with alterations in the activities of enzymes involved in the AsA-GSH cycle. Besides, chilling stress strongly enhanced anthocyanin levels in *S. officinarum* leaves [65]. Similarly, it was shown that transcript levels of genes involved in AsA metabolism, carotenoid biosynthesis and the phenylpropanoid pathway were significantly increased in *S. spontaneum* leaves subjected to drought stress [66]. In comparing two sugarcane cultivars with different salt stress tolerance, a proteomic analysis revealed that the tolerant cultivar had a higher abundance of proteins involved in non-enzymatic antioxidant mechanisms as compared to the sensitive one [67].

Fortunato et al., (2010) showed that the high cold tolerance of the Icatu genotype of coffee seedlings (*Coffea* genus) could be attributed to its efficient antioxidant response to cold conditions, involving increases in SOD and APX activities and concentrations of AsA and α-tocopherol [68]. Moreover, RNA-sequencing analysis revealed that the acclimation of *Coffea canephora* to drought stress involved upregulation of genes related to the metabolism of secondary antioxidant metabolites such as phenylpropanoids and flavonoids. The expression of genes involved in GSH metabolism was altered in the late response (i.e., after three drought cycles) to drought stress [69].

In tomato (*Solanum lycopersicum*), salt stress was associated with increased superoxide and H_2_O_2_ concentrations and an enhanced level of lipid peroxidation. This response coincided with increases in the activities of SOD, CAT and enzymes of the AsA-GSH cycle (APX, MDHAR, DHAR and GR). Furthermore, salinity significantly increased AsA and GSH concentrations, whereas carotenoid levels were reduced. The latter observation suggests ROS-induced damage to the photosynthetic machinery. Interestingly, the extent of ROS-induced damage upon salt stress was lower in the wild tomato relative *Solanum chilense* in comparison to cultivated tomato (*S. lycopersicum*), which can likely be attributed to the stronger salt-induced activation of the antioxidant machinery in the former species [70]. Upon drought stress, the activity of enzymes involved in the shikimate pathway and the concentrations of phenolic compounds such as caffeoylquinic acid derivatives, quercetin and kaempferol decreased in sensitive *S. lycopersicum* cultivars, whereas the opposite was observed in their tolerant counterparts. This emphasizes the importance of polyphenols in conferring drought tolerance in tomato [71].

Upon drought stress, chlorophyll and carotenoid concentrations in tobacco (*Nicotiana tabacum*) significantly decreased, whereas ROS levels and the extent of lipid peroxidation were strongly enhanced. This again points towards stress-induced damage to the photosynthetic machinery. Activities of SOD, POD, APX, CAT and GR and AsA, GSH and total phenol concentrations significantly increased to counteract drought-induced ROS production. This activation of the antioxidant machinery upon water deficit was even more pronounced when plants were inoculated with arbuscular mycorrhizal fungi or when they were supplemented with phosphorus, emphasizing the importance of associations with micro-organisms as well as soil characteristics in plant responses to stress conditions [72]. Tolerance of tobacco plants to As exposure is also conferred by antioxidant defense mechanisms as the As-tolerant genotype *N. tabacum* cv. ‘Wisconsin’ generally displayed higher phenolics, AsA and GSH concentrations in comparison to the As-sensitive genotype *Nicotiana sylvestris*. Antioxidant enzyme activities showed opposite responses between roots and leaves of both genotypes. Whereas APX, GST, and POD activity decreased in leaves of the tolerant genotype, they increased in leaves of the sensitive genotype. The opposite was observed for CAT. These data strongly indicate the organ specificity of antioxidant responses in plants, underlining the importance of studying responses in both organs [73].

The importance of the antioxidant defense system in plant protection against a broad range of stress factors is further evidenced by the fact that exogenous application of antioxidants such as AsA and GSH increases plant tolerance to several stress conditions [74,75,76,77]. Alternatively, plant stress tolerance can be enhanced via transgenic methods to improve their antioxidant potential. As reviewed by Broad et al., (2020), engineering of crops with an enhanced AsA concentration is used as an approach to increase crop tolerance to a variety of abiotic stresses. This can be achieved by (1) increasing the expression of genes involved in AsA biosynthesis, (2) increasing the expression of genes responsible for AsA recycling and (3) manipulating factors that influence AsA levels, such as transcription factors regulating the expression of genes in the AsA biosynthetic pathway [78].

### 3.3. Plants under Harsh Environmental Conditions as a Source of Antioxidants

Plants specifically growing in harsh environments are often characterized by increased levels of specific antioxidant compounds. Excellent examples are extremophiles that display an extraordinary ability to withstand unfavorable environmental conditions, such as salinity (halophytes), drought (xerophytes) and metal stress (metallophytes). For instance, the halophyte *Limonium virgatum* was shown to contain high concentrations of polyphenols such as tannins [79]. Similarly, *Anastatica hierochuntica*, an extremophile from the Brassicaceae family displaying increased resistance against heat stress, low nitrogen levels and salt stress, was characterized by increased concentrations of AsA and DHA in comparison to *Arabidopsis thaliana*. The same was observed for *Eutrema salsugineum* [80]. With regard to metal exposure, concentrations of several non-enzymatic antioxidants including carotenoids, phenolic compounds and AsA were higher in a metalicolous ecotype of *Silene vulgaris*, collected from a metal-contaminated site, in comparison to a non-metalicolous ecotype of the same species originating from a non-contaminated area. Interestingly, the metalicolous ecotype did not display an enhanced tolerance to salinity, highlighting the stress-specificity of plant defense mechanisms [81]. Due to their high antioxidant concentrations, extremophiles could be interesting sources for the discovery of new bioactive compounds [79].

Since plants in Cuba are frequently subjected to harsh environmental conditions (e.g., high temperature, drought, high light, salinity, nutrient-poor soil condition…), they are probably an underexploited source of antioxidants. Indeed, a few endemic Cuban plant species are already known for their antioxidant and bioactive properties, as summarized in Table 2. As such, conservation of these endemic species and further exploration of the antioxidant potential of other Cuban endemic plant species is of major interest.

## 4. Plant Nutritional Value for Human Health

Similar to their functions in plant protection, balanced levels of ROS and also reactive nitrogen species (RNS) play essential roles in the human body for normal development and physiological functioning on one hand, but also for protection against pathogens on the other hand. The in vivo antioxidant system can control the concentrations of ROS and RNS, thus maintaining the cellular redox equilibrium to allow oxidative signaling during lifespan. Nevertheless, excessive production of ROS and RNS as a result of pathophysiological cell metabolic processes, but also of many environmental abiotic stress factors such as radiation and environmental toxins, may trigger DNA damage and lead to protein and lipid oxidation. This may result in oxidative stress that underlies non-communicable chronic diseases [22,93,94]. Therefore, it is essential that the human antioxidant system can function in an optimal way to support human health. Antioxidants can be classified according to their source, i.e., endogenous synthesis of enzymes and some small molecules, such as GSH and coenzyme Q, as well as exogenous intake through our diet of mainly plant-derived phenolics, flavonoids, phenolic acids, carotenoids, vitamins and minerals.

### 4.1. Plant Secondary Metabolites with Antioxidant Activity: In Vitro versus In Vivo

There is no consensus on the best system to classify plant secondary metabolites. Categories based on the biosynthetic origin are most widely used and classify them into three main groups: (1) nitrogen- and sulfur-containing compounds, (2) terpenoids, (3) polyphenols and flavonoids [95]. A fast review indicates that all three groups include substances exhibiting antioxidant activity. The most prominent plant secondary metabolites with antioxidant activity show a common moiety. This consists of an aromatic ring linked to an atom with at least one unbound electron pair, being oxygen (most common and important), sulfur or nitrogen. Nitrogen and sulfur-containing compounds are the group less reported as antioxidant. Considering the bibliometric analysis developed using the Scopus online database and the keywords “antioxidant” and “antioxidant plus nitrogen and or sulfur compound”, these metabolites barely reach 5.41% of the hits (See Table 3). Non-protein plant nitrogen compounds (alkaloids) represent the group of metabolites mostly explored as phytochemicals, but only 4.33% as antioxidant. Two main reasons could explain this fact: the huge amount of other pharmacological activities inherent to this kind of metabolites and the relative infrequent presence of an active phenol group. Nevertheless, in the absence of a hydroxyl group, the redox reaction occurring during ROS neutralization generates benzylic radicals (less reactive than their phenoxy analogous) that are stabilized with the help of an electron pair from a neighboring nitrogen atom. As such, electron delocalization across the rest of the conjugated system is extended [96,97] and as a consequence its antioxidant activity. Terpenoids and especially essential oils received particular attention for their antioxidant activity (5.20% in the bibliometric study). Nevertheless, most of them rank as medium/low antioxidants, but once again, the most active compounds contain a phenol moiety. That is why eugenol, thymol and carvacrol often appear in different studies [98,99]. Other terpenoids with relative high antioxidant activity are the tetraterpenoids, carotenoids and xanthophylls. In the bibliometric study, they reach an 8.49% hit score within antioxidants. These compounds are lipophilic and have no aromatic ring, but their antioxidant activity is supported by a large number of conjugated double bonds that can stabilize the electrons donated.

**Figure 2:**
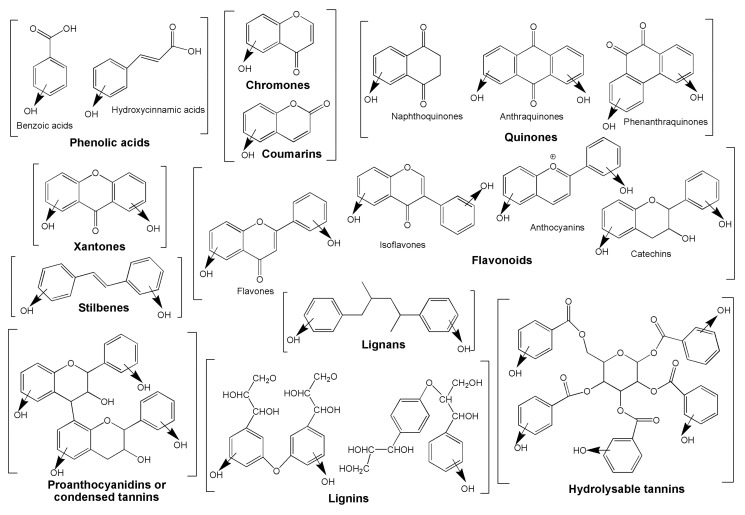
General chemical structures of the main classes of phenolic compounds and their derivatives.

As expected, polyphenols are the group of metabolites that received the most attention from the international community having by definition at least a phenol group in their basic structure and representing almost three quarters of the papers published on antioxidant potential in the bibliometric study (73.58%). Using a simple chemical classification system, they are divided in subgroups: phenolic acids (benzoic and hydroxycinnamic derivatives with C6-C1 and C6-C3 carbon backbone), hydrolysable tannins [(C6-C1)n], chromones and coumarins (C6-C3), quinones (naphthoquinones with C6-C4 and anthraquinones/phenanthraquinones, C6-C2-C6), stilbenes (C6-C2-C6), flavonoids (flavones, isoflavones, catechins, anthocyanins and others with C6-C3-C6), xantones (C6-C3-C6), proanthocyanidins or condensed tannins [(C6-C3-C6)n], lignans (C6-C3-C3-C6), and lignins [(C6-C3)n] (see Figure 2).

Independent of their structural diversity, this type of compounds shows an intrinsic “in vitro” antioxidant activity all possessing the aforementioned phenolic moiety. Each phenolic substituent will be able (in theory) to act as antioxidant due to its inherent low redox potential and hence its electron/proton donor capacity [100]. Therefore, metabolites with more than one hydroxyl group (polyphenols) can show more than one redox potential, varying in magnitude depending on the pH of the medium. Consequently, one could deduce that for a single compound more phenol substituents mean more antioxidant capacity, but evidently there are some limitations to this “rule”. Substituents adjacent to the phenolic moiety as well as the level of conjugation of the total structure can increase/decrease the reactivity of this group. First, phenols are irreversibly oxidized with the transfer of one electron and one proton into a phenoxy radical, which is more stable when another hydroxyl substituent is located in *ortho* and *para* positions. The *ortho* substitution pattern is more common, generating catechol groups which confer high antioxidant activity to the compounds with these characteristics. Secondly, substituents that contribute to the delocalization of the electrons through the resonant structures formed after the electron/proton transfer, also contribute to the in vitro effectiveness of polyphenols as antioxidants. Other chemical modifications that alter the redox reactivity of phenols are hydrophilic substituents like sugars that enhance the molecular solubility and promote the hydroxyl hydration in aqueous media, shifting the phenolic groups’ oxidation to more negative redox potentials. Bulky non-electroactive substituents may cause an unfavorable effect on the antioxidant activity due to the steric hindrance effect. A detailed explanation about phenol moiety oxidation mechanisms and factors that affect it can be obtained from the review recently published by Chiorcea-Paquim and collaborators (2020) [100].

As illustrated in Figure 2, complex polyphenols can reach high molecular weight by polymerization of their structure. Like this, tannins and lignins can easily accommodate dozens of phenol substituents in their structure, but at the same time such amounts of phenol groups hamper their activity modifying other chemical properties such as solubility. Tannins also show high reactivity when facing proteins, forming insoluble salts that inactivate these molecules. Considering the essential role proteins play in life, this property is in most cases more harmful than beneficial. Overall, simple phenols demonstrate low to medium antioxidant activity while on the other hand large molecules containing high numbers of phenols are useless due to undesirable properties for biological systems. Therefore, medium-sized polyphenol molecules with a number of phenol groups between three and seven are favored for their antioxidant activities. That’s why stilbenes [101], xanthones [102] and especially flavonoids [103,104] received a lot of attention from the scientific community. In fact, flavonoids are the largest subgroup of polyphenols in number of isolated molecules, but also receiving the most attention over the rest of the antioxidant secondary metabolites. By themselves, they are mentioned almost a quarter of the works published and indexed in the Scopus database independent of the nature or biosynthetic origin of the metabolite (see Table 3).

Despite all these in vitro characteristics that contribute to a high antioxidant potential, intake of antioxidants and/or nutraceuticals via the diet is limited to the ability of these compounds to be absorbed and distributed to the site of action. While a large number of hydroxyl groups point towards in vitro antioxidant activity, such compounds will easily violate the well-known rule of five for bioavailability and in consequence are not suited as drug candidates. This rule described for the first time in 1997 by Lipinski defines four points allowing absorption of these compounds: when molecules have (1) a maximum of five H-bond donors (hydrogens linked to a nitrogen or oxygen atom), (2) less than 10 H-bond acceptors (all nitrogen and oxygen atoms), (3) a molecular weight under 500 Da and (4) an octanol-water partition coefficient under the value of five [105]. This rule has been subjected to progressive updates even by the same author, but the essential parameters are maintained [106]. Therefore, this brings us back to simple substances with a limited amount of phenol moieties that are generally synthesized in larger quantities.

Due to the huge diversity of polyphenols, it is almost impossible to relate the diverse factors affecting the bioavailability of each class of these compounds. Besides the chemical structure of the compound, also the interindividual variability in health responses to this compound should be considered. For the latter, focus is on the functional status of the body like gender, aging, polypharmacy, prevalence of ailments on the intestinal tract, difficulties in nutrient absorption, dietary habits, etc. [107,108,109]. In case of flavonoids, we just want to highlight the most relevant in order to illustrate how complex it is to establish a parallelism between the in vitro and in vivo antioxidant activity.

The time required to absorb and distribute the flavonoid intake varies from 0.5 to 7 h depending on the chemical nature of the compound. Additionally, a low proportion (under 15%) is absorbed in the small intestine and then metabolized into different derivatives [110]. The kind of sugar moiety is the first characteristic that affects the time and extent of absorption. The linkage with sugars increases their water solubility and limits passive diffusion. Therefore, some authors affirm that glycosylated flavonoids initially undergo hydrolysis [111]. Lactase phloridzin hydrolase (LPH) present on the brush-border of small intestine epithelial cells is one of the suggested enzymes involved in this process. Alternatively, cytosolic β-glucosidase (CBG) that demonstrates a broad specificity towards polyphenols can perform this reaction, but in order to do this, polyphenols should be transported into epithelial cells, possibly by the active sodium-dependent glucose transporter SGLT1. The relative contributions of ‘LPH/diffusion’ and ‘transport/CBG’ depend on the position and nature of the sugar. While glucoside and xyloside glycosides are hydrolyzed faster by both systems, rhamnoglucosides must be deglycosylated by microfloral rhamnosidases and β-glucosidases present in the colon, extending the time of absorption [112].

The assessments of antioxidant action performed by polyphenolic compounds include both cell-free and cell-based in vitro assays and animal models as well. The in vitro models based on the free radical scavenging potential (e.g., ABTS, DPPH assays) offer prior information on how polyphenols lead to radical degradation, thus hypothesizing their antioxidant functions to combat oxidative stress in a biological system [24,113]. Other in vitro tests include enzyme inhibition, prevention of oxidative DNA impairment, reduction of intracellular ROS/RNS levels, anti-inflammatory and anti-proliferative activities. In the case of in vivo evaluation, the endogenous antioxidant enzyme estimations, the pro-inflammatory mediators release and the lipid peroxidation assay are some of the main tests performed [114,115]. Nevertheless, today a well-established methodology where different in vitro and in vivo assays can be combined is highly encouraged in order to evaluate the antioxidant potential of plant bioactive compounds.

### 4.2. Plant Medicinal and Nutritional Value of Antioxidant Candidates

In living organisms, ROS and RNS are oxidant substances normally produced in cell metabolism and participating in several cell protecting mechanisms. However, unregulated or unbalanced levels between free radicals and endogenous antioxidants may often provoke abnormal cellular processes defined as oxidative stress [116,117]. In this sense, oxidative stress can lead to pathological disorders defined as non-communicable chronic diseases, such as arthritis, asthma, cardiovascular dysfunction, autoimmune and neurodegenerative diseases, carcinogenesis, and diabetes [94,107].

In general, antioxidants are defined as substances able to reduce the oxidation [116,118]. A huge number of plant active chemicals have been identified as natural antioxidants during the last decades. Fruits, vegetables and non-edible plant parts are considered natural factories containing antioxidant phytochemicals able to maintain the oxidative balance, thus avoiding the ailments prevalence [21,119,120]. Antioxidants and phytonutrients present in vegetables, fruits and cereals are routinely consumed as natural diets [121], because they are safe and provide a myriad of health benefits. In the case of medicinal plants, natural antioxidants can also be incorporated into the body through traditional preparations like decoction, infusion or other herbal remedies [122]. Among the bioactive compounds present in plants and studied because of their antioxidant potential, vitamins, carotenoids and polyphenols are the most interesting health-promoting agents. It has been demonstrated that they may prevent lipid oxidation, inhibit the oxidation of some crucial enzymes and the uncontrolled release of pro-inflammatory cytokines, hence preserving normal cell functioning [123,124,125,126].

At present, there is a particular interest in polyphenols because of their presence in many natural resources, their chemical variety and their participation in crucial cell functions [127]. Naturally occurring polyphenols are widely distributed in almost all kind of plants and other organisms like fungi. They display significant effects on the reduction of pathological inflammation, liver and neurologic damage protection, and they have also been associated with lower risk of metastasis in cancer patients [22,128,129,130]. Nevertheless, the in vivo bioactive effects of polyphenols depend on their respective intake, absorption, metabolism, and bioavailability. As their absorption and blood distribution are rather low and at the same time elimination from plasma very fast [131], the consumption of these natural products should be on a daily basis during feeding and/or by nutraceutical supplements. This necessity of regular natural polyphenol consumption as well as the fact that they can change the food organoleptic properties and as a consequence the food quality, offers a link between medicinal plants and dietary supplements.

The antioxidant activities of polyphenols absorbed in mammals are exerted by different mechanisms. They can directly function as an antioxidant metabolite, i.e., as electron donor, as described before, but phenolic compounds may also participate in the regulation of enzyme functions (signal transduction) to maintain the oxidative balance. In this way, polyphenols exert their effect through the modulation of the nuclear factor erythroid 2-related factor 2 (Nrf2), a key transcription factor of the cellular antioxidant stress system. They can increase the activity of antioxidant enzymes like SOD, glutathione peroxidase and CAT to prevent oxidative damage, such as lipid peroxidation. On the other hand, polyphenols can also reduce or inhibit enzymes involved in free radical production such as NADPH oxidase, xanthine oxidase, lipoxygenase, monoamine oxidase and inducible nitric oxide synthase in order to prevent excess ROS production leading to oxidative damage, but at the same time ensuring oxidative signaling [24].

#### 4.2.1. Cuban Plant Antioxidants and Their Therapeutic Potential

The Caribbean region is recognized worldwide for its vast plant biodiversity including food and medicinal plants species [132,133]. Ethnobotanical reports validate that many native Caribbean herbal medicines are often used by the local people as traditional preparations to mitigate pain, combat infections and to treat chronic illnesses [134,135]. Nevertheless, the Caribbean region still contains a large number of plants unexplored and untapped from a therapeutic point of view. Cuba, as it was mentioned before, is considered one of the countries with the highest biodiversity worldwide, where more than half of the vascular plant species are native [136]. An extensive list of Cuban plants, endemic or not, has been incorporated in the National Guideline of Phyto-Medicines and Bee-Medicines (Formulario Nacional Fitofármacos y Apifármacos, 2010). This demonstrates the importance that the Cuban Health System confers to natural medicine. However, the enormous therapeutic potential of the flora associated with several plant-derived traditional formulations still lacks scientific evidence. Therefore, an increase in scientific studies to achieve this goal is justified. Accordingly, more Cuban plants and their bioactive extracts and compounds are being characterized and evaluated in order to explore and validate their pharmacological potential.

The major Cuban health problems are often associated with an inadequate lifestyle like a poor and unbalanced diet, physical inactivity, alcohol consumption and smoking. Consequently, there is a prevalence of cardiovascular diseases, hypertension, diabetes, obesity and cancer [137,138,139,140]. Therefore, many of the studies with Cuban plants are driven to elucidate the role of those active extracts and substances that can reduce the negative impact of these non-communicable chronic diseases [141]. In this review, a brief summary of several important biopharmaceutical studies on endemic as well as on more widespread plants is provided with a focus on a specific area within Cuba.

The Siboney-Juticí Ecological Reserve is located in the southeastern of Cuba, approximately 10 km southeast of the city of Santiago de Cuba [142]. The Reserve’s area is 20.8 km^2^. It retains all of its original terrestrial habitats, with coastal and precoastal xeromorphic scrub and semideciduous microphyll forest being the most abundant. In addition, three other habitats are present: mangrove stand, sea-grape woodland, and rocky-coastal vegetation complex. The Reserve has not been altered substantially by human activity. Interestingly, it harbors about 750 plant species including 159 endemics. This means that about 5.0% of Cuba’s total endemic vascular plants thrive on only 0.01% of the country’s surface area. Considering the dry and harsh environmental conditions, combined with the insularity of Cuba, a specific flora evolved with high chemical and pharmacological potentialities.

*Adelia ricinella* is a tropical tree member of the Euphorbiaceae family with a restricted distribution in Cuba and the Caribbean region, but with an abundant growth in the Siboney-Juticí reserve. Its folkloric use as a palliative for inflammation, fever and pain calls for scientific attention. Recently, a preliminary report showed the in vitro antioxidant potential of ethanolic and aqueous crude extracts from aerial parts of this species [143]. The flavonoid-enriched extracts were able to inhibit the ABTS radical in a concentration-dependent manner more strongly than AsA. An analytical phytochemical screening revealed the presence of a blend of twelve flavonoids derived from luteonin and apigenin (Berenguer et al., personal communication), compounds with antioxidant and anti-inflammatory activities [144,145]. Nevertheless, despite the empiric therapeutic knowledge accumulated, the high consumption of its biopreparations (decoctions, infusions, etc.) and the preliminary experimental results obtained, more scientific evidence is required to corroborate the antioxidant profile of this plant and it specific influence on the reported pathologies.

*Excoecaria lucida* (basionym, *Gymnanthes lucida*) is a bush distributed on the Caribbean islands, south of the United States and Central America and used in the Cuban culture because of its beneficial properties to combat asthma, infections and to alleviate toothaches [146]. For the first time, a bioassay-guided fractionation was reported for this plant species and showed ellagic acid, a natural dietary polyphenol, 3,3′,4′-tri-*O*-methyl ellagic 4-*O*-β-d-glucopyranoside acid and corilagin, as main compounds. Other polyphenol-like substances isolated from this plant were five coumarins with scopoletin and ayapin as the main components for this group [147,148]. Ellagic acid has been validated to contain good properties to scavenge in vitro radicals as well as exerting medicinal benefits. This was demonstrated through experimental studies involved in actions to reduce oxidation, inflammation, cell proliferation, and angiogenesis [149].

The Cuban endemic tree, *Zanthoxylum pistaciifolium*, better known as “palo vencedor”, naturally grows in the high and dry southern coasts located in the eastern region of the island and there is a high incidence in Siboney-Juticí Ecological Reserve. The traditional knowledge supports its use to treat lung infections and cold [150]. However, only a few experimental studies explored the medicinal spectrum of this plant until today. Aqueous, ethanol and n-hexane extracts obtained from the dried leaves were evaluated to determine the main secondary metabolite composition as well as its antimicrobial potential. Alkaloids, tannins and flavonoids were mainly identified in the ethanolic extract. Further partitioning by successive extractions with different polar and non-polar solvents and spectroscopic analysis of the phytochemicals highlighted the presence of especially the skimmianine alkaloid (main compound), associated with in vitro trypanocidal activity [151]. This alkaloid also has analgesic, sedative, anti-inflammatory and anticarcinogenic activities [152], but relative low antioxidant activity [153], probably because its hydroxyl substituents are blocked forming methoxyl-derivatives. Nevertheless, the high concentration of polyphenols in this plant suggests a high antioxidant potential, but further research is needed.

Another medicinal plant growing in the reserve is the species *Croton linearis*. This aromatic shrub widely distributed in the Caribbean region, is empirically used by the Cuban population as an analgesic to reduce fever and to treat infections [154]. In the *Croton* genus, the therapeutic effects have been mainly attributed to LMW compounds such as alkaloids, terpenoids and flavonoids [155]. However, little information is available about the *C. linearis* species, its phytochemical composition and pharmacological potential. In this sense, García et al., (2018, 2019) reported that the antimicrobial potential of this plant may be associated with the presence of alkaloids, flavonoids and essential oils [155,156]. Following a bioassay-guided fractionation, the studies identified seven compounds, which were for the first time isolated and characterized in this species, with alkaloids and flavonoid-derived substances among them. In summary, some of the active constituents from *C. linearis* showed in vitro activity against *Leishmania infantum* (alkaloid reticuline) and *Trypanosoma cruzi* (flavonoid glycoside isorhamnetin-3-*O*-(6″-*O*-*p*-*trans*-coumaroyl)-β-glucopyranoside). Until now, no study exploring the antioxidant potential has been performed yet. However, the anti-protozoal action observed, could be closely correlated with the antioxidant capacity of the isolated isoquinoline alkaloids (benzylisoquinoline and aporphine) [157]. Indeed, the free radical scavenging capacity of this group of compounds is related to its structural characteristics, which has been well described in the literature. Most aporphine and benzyl-tetrahydroisoquinoline alkaloids have multiple phenolic hydroxyls on the benzene ring and are expected to scavenge reactive free radicals at least by generating thermodynamically and kinetically stable phenoxy radicals [158]. However, generally, aporphine alkaloids exhibit higher antioxidant activity than the latter even without hydroxyl groups. The reason may be dual: (1) increased spin delocalization of phenoxy radicals in the plane biphenyl configuration in aporphine leads to a more stable structure after accepting a radical and (2) the nonphenolic analogues presumably act through the delocalization of the neighboring nitrogen lone electron pair.

In addition to the research performed on plant species in this specific Cuban region, some studies on plants, present worldwide but also in Cuba, with potential antioxidant activity have been also performed. Two of the food plants most popularly known by Cuban inhabitants are mango (*Mangifera indica*) and tamarind (*Tamarindus indica*). Although both trees are not endemic plants from Cuba and the Caribbean, they are geographically broadly spread on the island and grow in different locations. The fruits from these tropical trees contain many nutrients and are often eaten fresh, after homemade preparations or industrially processed. All plant parts are used because of their medicinal value documented in several scientific reports [159,160].

In the case of tamarind, fruits and leaves are edible and can be used in salads, stews and soups [161]. The nutritional value of tamarind leaves is supported by fat, fiber, carbohydrate and vitamin contents. Bioactive compounds like flavonoids and other polyphenols have been found in the leaves [162] with e.g., high levels of total phenols, mainly of the flavonoid type in an ethanolic extract [160]. Using an optimized extraction protocol for *T. indica* leaves, the pharmacological potential could be increased [161]. Leaf extracts demonstrated a strong antioxidant activity in human blood cells reducing ROS levels and protecting the erythrocyte membrane against H_2_O_2_-induced damage [163]. The antioxidant profile was also demonstrated in cell-free experiments, confirming the potential of this plant as an antioxidant supplement [164]. In general, these studies have contributed to the valorization potential of tamarind leaves and therefore, tamarind may become a good natural resource for novel and promising nutraceutical and cosmeceutical compounds.

The tree *M. indica* is especially distributed in the eastern region of Cuba, housing some particularly sweet mango varieties. The ethnomedicinal practices refer to the use of mango stem bark decoctions for the treatment of menorrhagia, scabies, diarrhea, syphilis, diabetes, cutaneous infections, and anemia [159]. A water extract derived from *M. indica* stem bark is the most studied natural antioxidant derived from plants present in Cuba. It consists of a blend of antioxidant chemical components such as polyphenols, terpenoids, steroids, fatty acids, microelements and the xanthone mangiferin, as the main component [165,166]. This extract prevents the TNF-α-induced IκB degradation and hence binding of NF-κB to DNA, reducing the gene expression of some mediators and enzymes implicated in inflammation, pain and oxidative stress processes [166]. Additionally, it diminishes ROS-induced liver damage in rats, inhibits the brain phospholipid peroxidation and DNA damage induced by iron and copper ions [167,168]. Because of its strong antioxidant potential, the extract of the tree bark was optimized and commercialized and is known under the trademark Vimang^®^. This product is approved and registered by the Cuban health regulatory agencies and the Cuban Industrial Property Office, Ministry of Science, Technology and Environment (OCPI, CITMA) as a nutritional supplement, phytomedicine and cosmeceutical [166,169]. Vimang^®^ is dispensed as different pharmaceutical formulations like raw dried material, tablets, creams and extracts, being the best and most selling natural antioxidant in the Cuban medicinal market. Although *M. indica* is a worldwide tree, the results obtained in studies and the experience accumulated by ethnobotanic, experimental and clinical studies for years in Cuba will allow the development and valorization of new phytomedicines and natural supplements elaborated from endemic plants but widely distributed all over the Cuban island.

### 4.3. Advances in Natural Antioxidant Formulations: A Potential to Valorize Cuban Nutraceuticals and Cosmeceuticals

Like pharmaceuticals, nutraceutical compounds are also categorized as therapeutically active substances able to prevent and cure several disorders. Nutraceuticals are not conventional foods, but they are delivered in a pharmaceutical form like tablets, capsules, powder, etc. [170]. However, there are still some discrepancies to classify them or to correctly define them as nutraceuticals because of different regulations in which such kind of compounds can only be registered as functional foods and nutritional or dietary supplements [171]. Nowadays, it is largely accepted that the nutraceutical concept involves formulations that contain certain pure active ingredients or substances isolated from food sources, herbal products and dietary supplements, which have a proven therapeutic effect when they are regularly consumed [28,172,173]. In other words, nutraceuticals are pharmaceutical formulations that by their periodic consumption exert both nutritional and therapeutic effects. Nonetheless, other terms are indistinctly used in the scientific literature to refer to bioactive chemicals present in plants and foods, some of them have been named phytonutrients, phytodrugs, pharmafoods and phytocomplexes [174]. Plant-based nutraceuticals, mainly secondary metabolites like polyphenols, have demonstrated to exhibit therapeutic and disease-preventing effects in animal models and human trials. Cardiovascular diseases, diabetes, cancer, neurodegenerative disorders like Alzheimer’s and Parkinson’s diseases, are currently receiving more significant attention because of the underlying relation with oxidative stress [62,127,175].

Nowadays, there is also an increasing interest to produce cosmeceuticals with corroborated physiological benefits based on natural active ingredients from plants. They are mainly used as external applications such as skin nourishment and protection. The term “cosmeceuticals” refers to a hybrid functionality combining the principles of cosmetics and pharmaceuticals intended to provide health benefits [29,176]. Among the medicinal properties of cosmeceutical usages are antioxidant action, anti-inflammatory and analgesic activities, wound healing, anti-aging, etc. [177,178]. Similar to pharmaceuticals and nutraceuticals, vitamins, essential oils, carotenoids and mainly polyphenols are the most investigated and promising biologically active compounds for cosmeceuticals with dermatological applications and skin care [176,179,180]. Flavonoids and their derivatives have shown to possess external anti-inflammatory and anti-irritant activities mainly associated to the reduction of pro-inflammatory mediators. They are also proven solar protectors, reducing the harmful effects that free radicals (generated by the incidence of ultraviolet light) cause on the skin epidermis, accelerating the aging process and the appearance of undesirable spots and wrinkles. Supplementation with flavonoids improves the capillary fragility and it also exerts anti-microbial actions against bacteria and fungi, thus protecting the skin [181].

The Cuban national market related to nutraceuticals, dietary supplements and cosmeceuticals is at present limited despite the existing regulations to register plant-based medicines. Moreover, this new potential ‘medicinal’ source of products is not only restricted to plant derivatives, but also other natural sources such as edible mushroom and microalgae that are also underexplored and underexploited. Besides the vitamin-rich dietary supplements that are barely available, the *Spirulina platensis* and *Moringa oleifera* nutritional supplements and the aforementioned Vimang^®^, represent the best-known functional products commercialized and registered as nutraceuticals and cosmeceuticals in Cuba [166,182,183,184]. Nowadays, many efforts are intended to raise a market based on nutraceuticals and cosmeceuticals taking advantage of the Cuban biodiversity richness, especially when there is an accelerated increase in diseases associated with an inadequate lifestyle and unhealthy diets. Increasing efforts in the isolation and characterization of new secondary metabolites (i.e., polyphenols) from food and plant sources, and its subsequent evaluation and formulation as nutraceutical, food supplement and cosmeceutical presume an interesting assay-guided methodology. This must include not only studies on the design and stability of pharmaceutical formulations but also a well-structured platform for the development of in vitro and in vivo experiments to measure their bioavailability and efficacy, thus guarantying their therapeutic benefits. Cuban plants are natural product factories, housing uncountable new candidates for drugs and novel formulations. Nevertheless, most of the ethnobotanical information supporting the plant curative effects needs to be confirmed by a robust phytochemical, pharmacological and toxicological screening platform.

## 5. Sustainable Production of Plants/Crops with Improved Antioxidant Potential

**Figure 3:**
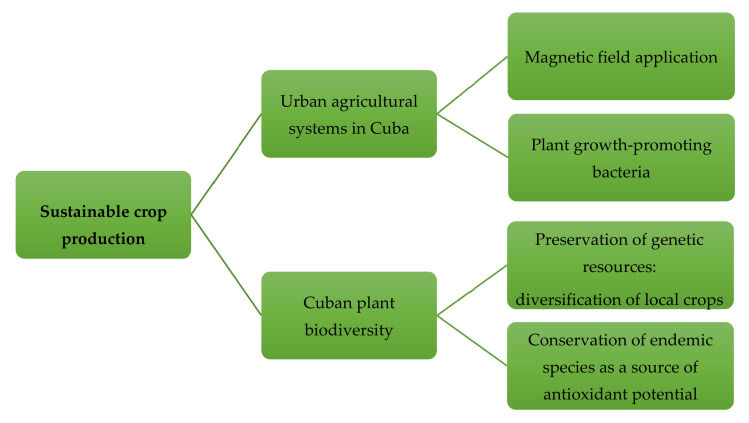
Promoting sustainable agriculture in Cuba based on agroecological practices and using Cuban plant biodiversity as a natural resource.

Environmental changes and anthropogenic activities have deeply impacted ecosystems worldwide and also in Cuba, with repercussions on agriculture. In addition, agricultural practices have been directed towards mass production for a selection of crops leading to the loss of soil quality and mineral impoverishment as well as a loss of genetic diversity [30]. Facing the extreme events due to climate change and the vulnerability of agriculture to these alterations, diversity in genetic resources will promote resilience of the agricultural systems, and buffer social and economic shocks that might arise from concentrating on fewer crops [185]. In addition, plant biodiversity harbors a reservoir of secondary plant metabolites and based on their relevance for human health and plant protection, they have been already studied and exploited to improve crops and food products [186]. Therefore, on one hand more research is needed to develop new strategies for sustainable agriculture to improve crop yield and quality, without exhaustion of natural resources. On the other hand, improving our knowledge on Cuban plant biodiversity and their antioxidant potential can offer new strategies for (1) diversification of agricultural systems via the cultivation of local nutraceutical crops and herbs and (2) nature conservation policies (Figure 3).

### 5.1. Urban Agricultural Systems in Cuba: An Unexplored Niche of Sustainable Agricultural Practices to Improve Nutritional Quality of Cultivable Crops

The production of vegetal foods in developing countries combining high crop productivity with high food nutritional quality, through sustainable agricultural practices, should constitute a substantial part of governmental strategies to safeguard food security. The use of strategies for biofortification of economically valuable crops has gained attention of the research community. The term ‘plant biofortification’ refers to enhancement of bioavailable nutrients and secondary metabolites like antioxidants in tissues of vegetal foods, by using traditional technologies like plant breeding, genetic engineering and plant fertilization [187,188]. Nevertheless, crossbreeding techniques are expensive, as well as time-consuming and imprecise, as the process requires successive crosses and back-crosses for several generations. In addition, parents must exhibit high genetic variability and heritability potential of genetic determinants [189]. Moreover, genetically modified crops are not well accepted as foods by several human communities [188] and must face the regulatory constraints on genetically modified organisms [190]. On the other hand, use of synthetic chemicals to enhance antioxidant compounds and nutritional quality of fruits and vegetables is discouraged, because of the potential threat to the consumer’s health and the possible deleterious effects on the environment [187]. Therefore, biofortification of crops with bio-fertilizers and agroecological practices has major advantages over these technologies.

In the twentieth century, Cuba experienced a major national food crisis due to the reduced availability of fertilizers and pesticides. Food production was at critical levels; uptake of calories, proteins and vitamins in the Cuban population decreased over more than 40 percent. Emergent and re-emergent chronic and infectious diseases, associated with malnourishment, increased and because of the lack of basic medicines, this strongly impacted on the human immune system [191]. In order to meet the basic food requirements of the Cuban population, the agricultural sector implemented a national program of urban and periurban agricultural production based on the application of sustainable, eco-friendly agronomic practices like organic amendments, bio-fertilizers and bio-pesticides [192]. The urban agricultural program renders more than 50% of plant-based foods consumed by the Cuban population like herbs, condiments, medicinal plants, fruits, and tuber crops [193].

Urban and periurban agricultural systems developed in Cuba are a suitable platform to design and introduce innovative, sustainable agronomic practices for economically valuable crops. Innumerable positive outcomes of scientific studies have been transferred to agricultural practice and extended to different productive models implemented into the program, such as organoponics, hydroponics, family gardens, cooperatives, small-scale farmhouses and big-scale governmental farms [191,194,195]. In order to optimize the growth conditions for sustainable crop production, several technologies, such as application of magnetic field and plant growth-promoting bacteria have been adopted in Cuban plant growth factories as well as implemented in in situ crop production.

#### 5.1.1. Application of Magnetic Field to Improve Seedling Performance and Crop Yield and Quality

One of the objectives of agriculture is the constant supply of vegetables. To achieve this goal, a high germination and survival rate of the seedlings is of vital importance. To stimulate germination rate, many factors are evaluated, including light, temperature, and chemical treatments, but treatments with magnetic field (MF) are often neglected despite their potential stimulating effect [196]. The MF technology can be directly applied on the seeds or on the irrigation water used for seedling growth. Poinapen et al., (2013) evaluated the effect of applying static MF on seeds in combination with intrinsic seed viability factors, such as relative humidity with regard to seed germination in *S. lycopersicum* (tomato). They found that the most pronounced effects of static MF treatment occurred mainly during seed imbibition rather than during later developmental stages [196]. Gonzalez Aguilera et al., (2016) investigated whether irrigation with MF-treated water had an influence on seed performance of *S. lycopersicum*. In comparison to the control group, they noted a substantial increase in seed germination (36%), plant height (97%), stem diameter (12%) and even the number of leaves (5%). By using the MF technology on the irrigation water, the seed germination process was accelerated and the development of tomato seedlings stimulated [197]. In another study on different crops, the effect of MF-treated water on seed germination and their survival was evaluated with a very positive outcome. These included bean (*Vicia faba*), tomato (*S. lycopersicum*), pumpkin (*Cucurbita maxima*) and cucumber (*Cucumis sativus*) [198]. The application of this technology accelerated the germination process in all these plant species, but the greatest survival was evidenced in bean, cucumber and tomato seedlings. Besides their higher survival rate, seedlings could be transferred earlier to other cultivation areas where they continued their growth. As such, production costs are reduced at this stage of agricultural work. Similar observations were made when coffee seedlings were cultured in vitro and MF treatment was applied [199]. Application of MF during the establishment, multiplication and acclimatization phases of growing coffee seedlings resulted in a stimulation of the photosynthetic activity and hence better functional capacity of the leaves in combination with decreased oxidative stress, leading to improved seedling growth. As this is a safe technology, application of MF might offer a solution to improve the in vitro plant growth environment that corresponds to the current requirements of environmental protection and agricultural development.

Besides improving seed germination and seedling performance of general crops, application of the MF technology to plants that are difficult to propagate, might be very useful. *Rosmarinus officinalis* (rosemary) is a model plant as nutraceutical due to the numerous antioxidants and secondary metabolites it produces under natural conditions. However, its seeds have a low viability, and under normal natural conditions their germination rate is very low. Furthermore, germination of rosemary seeds can take more than 35 days under certain conditions. By using an MF treatment (100–160 mT), a strong reduction in the germination time of the rosemary seeds was achieved [200].

The use of MF technology is not only limited to seed germination and seedling performance, but can also be exploited for in vitro production of plant tissues to obtain bioactive compounds. Indeed, it was shown that MF technology in combination with other plant growth regulators, such as plant hormones, stimulated the production of secondary volatile metabolites with antioxidant activity in *R. officinalis* calli [201]. In this way, in vitro MF treatment is a very powerful tool to valorize plants of interest that are found in isolated locations and that are present in limited numbers.

As seedling establishment is a first important criterion for successful agriculture, maintenance of crop yield and quality is the second one. The application of MF on irrigation water in agriculture is an important method with positive effects on physiological and biochemical aspects in different plants. A stimulation of growth and development has been evidenced in various phenological plant stages [199]. Currently there are various theories and hypotheses on the mode of action of this physical agent on plant growth and development [202,203,204,205].

A species of great agronomic and economic interest is tomato (*S. lycopersicum*), which is the second most consumed vegetable worldwide and is especially known for its nutritional benefits [206]. Tomato plants grown during 120 days and irrigated with MF-treated water using a drip irrigation system demonstrated an improved growth in comparison to control plants. Moreover, optimizing the magnetic induction (strength, duration, frequency) resulted in higher fruit weights. In follow-up experiments, both aqueous and ethanolic extracts were prepared from the tomato fruits to investigate whether applying MF-treated irrigation water affected their nutritional value. An increase in the content of the antioxidant AsA and of the secondary metabolites gallic acid, rutin and quercetin was observed in the extracts from fruits of plants irrigated with MF-treated water. With the use of an optimal magnetic induction, the synthesis of phenols and flavonoids was highly stimulated and the antioxidant properties of tomato fruits were increased. In general, the application of MF-treated irrigation water positively affects the presence of antioxidant compounds in tomato fruits, which can increase their nutritional value [207,208]. Moreover, the increased content of secondary metabolites can positively influence food production and the use of bioactive compounds for pharmaceutical purposes.

Similar to enhanced germination performance, improved production of plants with medicinal interest that are traditionally used by the Cuban population is of utmost importance. For example, *R. officinalis* (rosemary) was removed from the National Formulary of Phytopharmaceuticals of the National Health System, due to the lack of availability of the raw plant material. Therefore, several studies were carried out to evaluate the effects of MF-treated irrigation water on the production of this species. Growing *R. officinalis* under different magnetic induction regimes improved morphological traits such as stem and root length, as well as leaf area [209]. For a better understanding of the effects of MF-treated irrigation water on plant growth and development, the elemental composition in these plants grown under optimal magnetic induction was determined and a significant increase in calcium, potassium and magnesium was observed. Furthermore, an increase in qualitative nutritional and pharmaceutical properties was observed in MF-irrigated plants. Investigating the monoterpene metabolites in hexanolic extracts of *R. officinalis* L. revealed the presence of higher contents of linalol, citral and citronellal in plants irrigated with MF-treated water. The characterization of aqueous extracts highlighted an increase in different antioxidant compounds such as rosmarinic acid, camphor, α-terpineol, verbenone, endo-borneol, bornyl acetate and β-amirin in the plants irrigated with MF-treated water [210,211].

Also for other plant species, similar improvements in plant developmental as well as nutraceutical and/or pharmaceutical characteristics were observed when they were cultivated using MF-treated irrigation water. In *Origanum majorana* cultivated in these conditions, an increased stem length, a larger foliar area with an increased number of stomata, elevated concentrations of photosynthetic pigments, and increased secondary metabolites with antioxidant activity, such as phenols and flavonoids were noticed [212]. Green beet (*Beta vulgaris* var. cicla) and red beetroot (*B. vulgaris* var. rubra) are crops with a high potential as nutraceutical food, since they contain phytochemicals with considerable antioxidant activities that have beneficial effects on human health [213]. Application of MF technology during crop production resulted in an increased biomass in beet plants [198], which was also confirmed in another study where an increase of 94% in the root mass and 52% in the leaf surface of beet plants was noticed after irrigation with MF-treated water [214]. Higher biomass and fresh weight production were also observed for pepper and chickpea when plants were irrigated with MF-treated water [215]. These results clearly show the potential of MF technology in conventional irrigation systems to enhance the production of nutraceutical foods, as well as plant-derived pharmaceutical products with high antioxidant activity.

#### 5.1.2. Plant Growth-Promoting Bacteria Stimulate Plant Growth Performance in Economically Valuable Crops

Plant growth performance can also be improved by plant growth-promoting bacteria (PGPB) and numerous recent studies reported their beneficial effects on economically valuable crops by increasing their nutritional quality [187,188,216]. The use of PGPB that enhance mobilization of macro- and micronutrients from soils and consequently, their uptake by plants, increasing nutrient content in plant tissues, gave birth to the term ‘plant probiotic bacteria’ [217,218]. Biofortification of crops using PGPB relies on the microbial abilities to increase the bioavailability of nutrients, their influence on pH, organic matter and their role in nitrogen fixation as well as mineralization of nitrogen and phosphorous from organic and inorganic complex substrates present in the soil [219,220]. Consequently, a high content of these macronutrients in plant tissues improves the water status of these PGPB-colonized plants and facilitates solubilization of mineral elements like potassium, magnesium, calcium, iron, zinc, boron and manganese [221]. Co-inoculation of plant seedlings with *Bacillus*, *Pseudomonas* and *Rhizobium* enhanced the nutritional quality of tomato fruits [221], as well as common beans, chickpea, wheat and soybean [188]. Inoculating lettuce plantlets with *Bacillus methylotrophicus*, a gibberellin producer, enhanced the content of several essential amino acids and micronutrients that have a key role in human physiologic processes like the immune response by improving the efficiency of protein synthesis [219].

In the field of PGPB and their application as bio-fertilizers, commercial inoculants are formulated with *Rhizobium*, *Bradyrhizobium*, *Azotobacter*, *Gluconacetobacter* and *Pseudomonas*. Application of bacterial inoculants has been employed to improve plant growth and development, as well as crop productivity. Major PGPB-favored crops are sorghum, corn, rice, papaya, tomato and *Canavalia* and constitute a source of proteins and carbohydrates with direct contribution to improve protein-energetic nutrition to humans and livestock [222]. In addition, these PGPB-cultivated plant species are rich sources of essential nutrients and micronutrients beneficial to human health, e.g., tomato [223], corn [224] and rice [225].

In recent years, research studies on autochthonous PGPB developed in Cuba, revealed that several crops identified as functional foods because of their nutritious quality and antioxidant potential, respond very well to PGPB application, organic amendments and other eco-friendly agronomic strategies used in the urban agricultural system (Table 4). Inoculation of eggplant and pepper seedlings with *Brevibacillus borstelensis* B65, a phosphate-solubilizer [226], and rice seeds with diazotrophic strains of *Pseudomonas* AI05 and AJ13 [227], revealed that these autochthonous PGPB positively impacted plant growth and development as well as crop yield through improving nutrient availability and consequently, nutrient uptake by the plants. On the other hand, the effects of PGPB on stimulation of plant antioxidant production have mainly been studied in fruit trees, herbs and vegetables. *Bacillus*-like and Pseudomonads species enhance carotenoids, flavonoids, polyphenols and anthocyanins in fruits and ornamental plants [187,228]. Co-inoculation of amaranth plants with different *Bacillus* isolates significantly increased the content of essential amino acids in amaranth grains. Consequently, it improved the regulation of protein synthesis and the immune response in humans when consumed [229]. Several aspects of the mode of action used by PGPB to enhance antioxidant substances in plants are unclear. It seems that bacterial phytostimulation may trigger the production of essential oils in ornamental plants [230]. Moreover, plant-bacterial interactions display a plethora of mechanisms that involve routes of primary and secondary metabolism that are quite diverse, but also overlapping [188]. This suggests that bioprospection studies of PGPB constitute an interesting area that could render new knowledge contributing to the elucidation of new metabolic routes for plant antioxidants production and the discovery of new antioxidants. Autochthonous PGPB applied as inoculants could also be valorized with innovative applications in the field of nutraceuticals and medicinal foods. Whereas the application of PGPB as inoculants is a promising “green technology” for increasing crop yield and quality, more research is needed on the impact of PGPB on plant secondary metabolism and bioactive compounds [187].

### 5.2. Cuban Plant Biodiversity as a Source of Antioxidant Potential

In addition to the diverse agroecological cultivation methods to improve crop yield and quality, introducing native non-crop species into agricultural systems as a way to provide bioactive compounds and antioxidants has recently gained attention [35]. As many Cuban endemic plant species harbor antioxidant and pharmaceutical potential (Table 2, Section 4.2.1), this offers a great opportunity to obtain new cultivars well adapted to different edaphoclimatic niches. Integrating native plant species with potential nutraceutical and/or pharmaceutical compounds into agriculture can be facilitated using biotechnological and agroecological approaches.

To investigate whether non-model and non-crop plants, such as many Cuban endemic species that are highly promising in the search for bioactive compounds, can be valorized [166,169], it is essential to focus on their ultimate purpose as nutraceutical and/or pharmaceutical in order to optimize plant production. In vitro plant (tissue) production to generate large amounts of natural raw material can be optimized, but it is pivotal to take the cost-benefit ratio into account. To further extend crop production in an agricultural setting, the use of platforms enabling translational research is highly recommended [238,239,240,241]. Using this approach, the experimental setups range from strictly controlled in vitro experiments to environmentally realistic in situ experimental plots. The application of agroecological processes, such as the use of MF or PGPB, in controlled experimental settings can give positive outcomes, and is an interesting approach to investigate the mode of action at different biological organization levels. Nevertheless, the positive outcome needs to be confirmed when different environmental factors come into play, when plants are grown in open-field conditions. As current scientific studies mainly focus on the impact of applying one agroecological practice on a specific developmental stage of the plant, future research should also explore the benefits of combining different practices to address the specific needs to optimally grow these plants in the environment.

## 6. Conclusions

Cuba holds an extremely rich endemic plant biodiversity. This wealth in diversity is currently underexplored in terms of antioxidants and bioactive compounds mainly residing in plant secondary metabolites. This group of antioxidant phytochemicals is characterized by an enormous chemical diversity, which represents a gold mine of information both on the evolutionary adaptation to harsh environmental conditions and on the nutraceutical or pharmaceutical value of these plant species. Therefore, increased scientific attention for this research field is of utmost importance, not only to gain more knowledge on known antioxidant compounds, but also to discover new natural antioxidants. Subsequently, this can guarantee economically valuable products for human health. Biofortification strategies aiming to improve the nutraceutical and/or pharmaceutical value of plants will offer the opportunity to develop new enriched plant-derived foods and products. These could be useful either in the maintenance of human health or in the prevention or treatment of human pathologies. In addition, an immediate, increased scientific exploration of the antioxidant potential is needed as many Cuban endemic species are in danger of extinction. Integrating this scientific knowledge into ex situ valorization strategies emphasizes the need for in situ conservation of local species, which obviously requires incorporation in future nature management policies.

## Figures and Tables

**Table 1:** Plants species with antioxidant activity and their derived products used in Cuba (Dirección de medicamentos y tecnologías médicas, 2018).

Species	Natural Products
*Capsicum* sp.	Cream; Tincture
*Bixa orellana*	Oil extract
*Allium cepa*	Cream; Syrup
*Zea mays*	Syrup
*Passiflora incarnata*	Syrup
*Mentha piperita*	Dry drug; Fluid Extract; Syrup
*Citrus aurantium*	Tincture; Syrup
*Origanum vulgare*	Syrup
*Pinus caribaea*	Cream; Fluid extract
*Bidens alba*	Dry drug; infusion
*Calendula officinalis*	Syrup
*Allium sativum*	Tincture; Syrup; Tablet
*Zingiber officinale*	Tincture
*Musa paradisiaca*	Pediculicidal lotion
*Cymbopogon citratus*	Cream
*Salvia officinalis*	Cream; Fluid extract; Syrup
*Mangifera indica*	Cosmetic cream; Tablet; Aqueous extract
*Rhizophora mangle*	Tincture; Fluid extract
*Matricaria recutita* o *Matricaria chamomilla*	Mouthwashes
*Psidium guajava*	Dry drug; infusion
*Moringa oleífera*	Dry drug

**Table 2:** Cuban endemic plants investigated for their antioxidant and bioactive properties.

Endemic Species	Antioxidant Compounds	Bioactive Properties	References
*Jacaranda arborea*	Polyphenols and flavonoids (luteolin, jacarananone, tripterpens, ursolic acid and oleanolic acid)	Anticarcinogenic and sedative	[82,83]
*Maytenus cajalbanica*	Polyphenols		[84]
*Phyllanthus orbicularis*	Phenols and flavonoids	Photoprotective and antimutagenic	[85]
*Phyllanthus chamaecristoides*			
*Phyllanthus microdictyus*			
*Phyllanthus williamioides*			
*Scutellaria havanensis*	Flavonoids (wogonin)	Anti-inflammatory, anti-allergic, anxiolytic, neuroprotective, anticonvulsant, antithrombotic, anticarcinogenic, anti-arthritic, antisplasmodial, antiviral and antimicrobial	[86,87,88]
*Calophyllum rivulare*	Pyranochromanone acids and amentoflavone		[89,90]
*Erythroxylum alaternifolium var. alaternifolium*	Flavonols (quercetin-3-*O*-rutinoside and ombuin-3-*O*-rutinoside)		[91]
*Erythroxylum alaternifolium var. parvifolium*			
*Eugenia clarensis*	Phenols, tannins, triterpenoids, sterols, flavonoids, coumarins,	Sedative	[92]

**Table 3:** Most mentioned plant secondary metabolites associated with antioxidant activity after bibliometric analysis in the Scopus online database (https://www.scopus.com/).

TOPIC	Total of Documents	% of the Total
Antioxidants	455,065	100
Antioxidants/plant	114,148	25.08
**Antioxidant category**		**% of the Plant Total**
Alkaloids	4937	4.33
Sulfur compounds	1237	1.08
**Partial scores for group one**	**6174**	**5.41**
Terpene/terpenoid	6086	5.34
Essential oil	5931	5.20
Carotene/carotenoid	9689	8.49
**Partial scores for group two**	**21,706**	**19.03**
Polyphenol/phenol	49,267	43.16
Flavonoid	27,058	23.70
Tannin	6191	5.42
Coumarin	1487	1.30
**Partial scores for group three**	**84,003**	**73.58**

Hits are scored when all the words “defined in the search pattern” appear in the title, keywords and/or article abstract.

**Table 4:** Effects of plant growth-promoting bacteria (PGPB) on economically valuable crops cultivated in Cuban urban agricultural systems.

Crop Species (Scientific Name)	Plant Growth-Promoting Bacteria *		
	PGPB	Plant Growth-Promoting Effects	Reference
Tomato (*Solanum lycopersicum*)	*Bacillus* sp.	Seed germination and vigor; root development	[231]
	*Azospirillum brasilense*	Crop yield	[232]
Eggplant (*Solanum melongena*)	*Brevibacillus borstelensis* *	**Seedlings:** Germination index; shoot root length, biomass	[233]
		**Adult plants:** Number of flowers, leaves and fruits; fruit, crop yield	[226]
Sugar beet (*Beta vulgaris*)	*Brevibacillus borstelensis* *	Seed emergence; plant growth	[234]
Pepper (*Capsicum annum*)	*Brevibacillus borstelensis* *	**Seedlings:** seed emergence; biomass, root development	[233]
		**Adult plants:** Number of flowers, leaves and fruits; crop yield	[226]
Maize (*Zea mays*)	*Bacillus* sp.	Seed germination and vigor; root development	[231]
	*Burkholderia cepacia*	Seed germination, seedling growth	[235]
Rice (*Oryza sativa*)	*Burkholderia cepacia*	Seed germination, seedling growth	[235]
	*Bacillus* sp.	Root formation and development	[236]
	*Pseudomonas* sp.	Increased plant growth, dry biomass, total N	[227]
Common bean (*Phaseolus vulgaris*)	Consortium: *Bacillus subtilis*, *Lactobacillus bulgaricus, Saccharomyces cerevisiae*	Crop yield	[237]

* Plants grown in the presence of PGPB and organic fertilizers.

**Figure A1:**
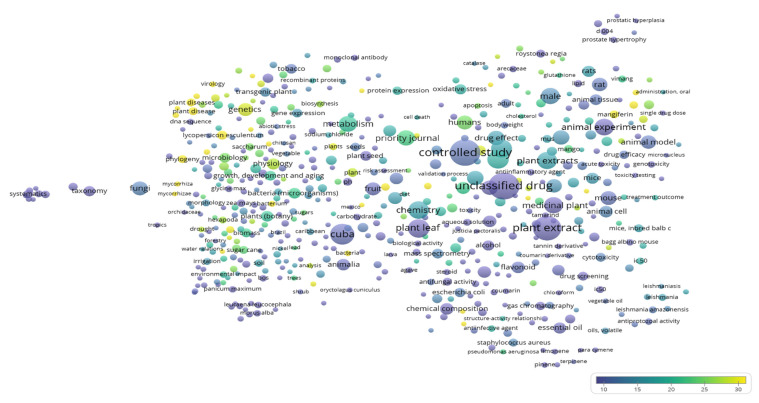
Term map for Cuban plant related publications. The bubble map visualizes 544 terms that appeared in at least 10 of the included publications. Bubble size indicates the frequency of occurrence of the words. Bubble color indicates the averaged citation count received by publications containing the word in their titles or abstracts.

**Figure A2:**
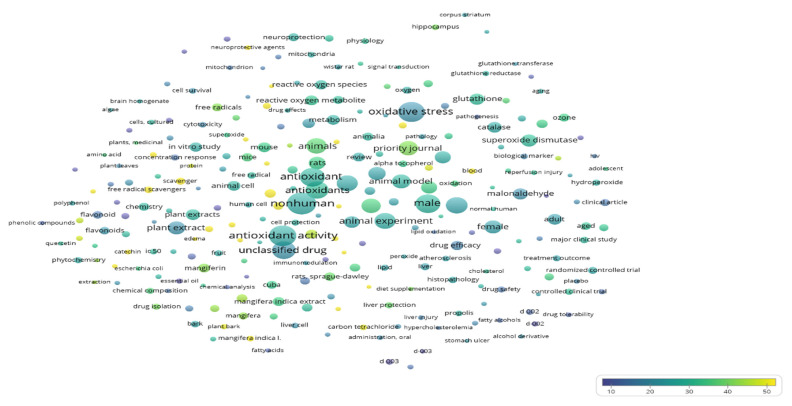
Term map for Cuban antioxidant related publications. The bubble map visualizes 258 terms that appeared in at least 10 of the included publications. Bubble size indicates the frequency of occurrence of the words. Bubble color indicates the averaged citation count received by publications containing the word in their titles or abstracts.

## Appendix A

A literature search using Cuban publications in journals indexed to Scopus with the keywords ‘antioxidant’ (=antioxida*; anti-oxida*) and ‘plant’ was performed in July 2020 as a basis to perform a VOSviewer analysis. It should be taken into account that there are studies published in Cuban journals that either are not indexed to Scopus or only have been recently. This leads to a certain number of publications being outside the scope of this literature search and therefore not accounted for.

These publications were imported into VOSviewer (CWTS, Leiden University, Leiden, The Netherlands) for bibliometric analysis. A co-occurrence analysis was realized taking all keywords into account presented in the papers, employing the full counting method and considering 10 as the minimum number of occurrences of a keyword. The VOSviewer software visualizes the results as a bubble map (van Eck & Waltman, 2010); two were generated. The first one focussed only on plant-related papers and the second one on antioxidant-related papers.

For the plant papers, a total of 3056 publications were identified and analysed. From that, a total of 14,741 keywords were reduced to 544 taken into account its number of occurrences (Figure A1). Topics with high citations included plant extract (272 occurrences), medicinal plant (124 occurrences), chemistry (142 occurrences), antioxidant activity (83 occurrences) and antioxidant (66 occurrences). Of the total number of publications (3056), 94 were specific to endemic species.

The second bubble map was constructed employing 699 publications related to antioxidants. From a total of 6079 keywords, only 258 were represented considering its number of occurrences (Figure A2). The most cited topics included oxidative stress (232 occurrences), antioxidant activity (242 occurrences), antioxidant (201 occurrences), antioxidants (170 occurrences), lipid-peroxidation (125 occurrences), plant extract (99 occurrences), mangifera indica extract (47 occurrences), mangiferin (45 occurrences) and flavonoids (32 occurrences). Of the total number of publications (699), 9 were specific to endemic species (Table 2).
